# Direct modulation of human GABA-A α_1_β_2_γ_2_ receptors by the endocannabinoid 2-arachidonoylglycerol: implications for cannabinoid-related ligands and limitations for anxiolytic drug development

**DOI:** 10.3389/fnins.2026.1813618

**Published:** 2026-05-13

**Authors:** Edgar Mixcoha, Anabel Flores-Zamora, Ricardo Hernández-Miramontes, Jorge Alberto Hernández-Calderón, Susana Martiñón

**Affiliations:** 1 Secretaría de Ciencia Humanidades Tecnología e Innovación - Instituto Nacional de Medicina Genómica, Mexico City, Mexico; 2 Facultad de Ciencias de la Salud, Universidad Anáhuac, Mexico City, Mexico; 3 Instituto Nacional de Psiquiatría Ramón de la Fuente Muñiz (INPRFM), Mexico City, Mexico

**Keywords:** 2-arachidonoylglycerol, allosteric modulation, anxiety, cannabinoid-related ligands, GABA-A receptor

## Abstract

Anxiety disorders are associated with impaired inhibitory neurotransmission mediated by γ-aminobutyric acid type A (GABA-A) receptors. Although benzodiazepines remain effective anxiolytics, their clinical utility is limited by sedation, cognitive impairment, tolerance, and dependence, prompting the search for mechanistically distinct GABAergic modulators. Among cannabinoid-related molecules, the strongest evidence for direct GABA-A receptor modulation concerns the endocannabinoid 2-arachidonoylglycerol (2-AG), which potentiates recombinant human α_1_β_2_γ_2_ receptors through residues located in the M4 helix of the β_2_ subunit. Here, we review the structural architecture, biophysical properties, and pharmacological profile of the human GABA-A α_1_β_2_γ_2_ isoform as the relevant molecular framework for evaluating this mechanism, while discussing the broader relevance of cannabinoid-related ligands and selected phytocannabinoids without assuming mechanistic equivalence. We further assess the hypothesis that 2-AG reaches the β_2_-M4 site through a membrane-access route and identify five conceptual barriers that currently limit translation of this mechanism into anxiolytic drug development: supraphysiological effective concentrations, unresolved synaptic-versus-extrasynaptic actions, uncertain subtype selectivity, incomplete validation of lipid-environment effects, and lack of clinical evidence linking this mechanism to anxiolysis in humans. We conclude that direct modulation through β_2_-M4 defines a mechanistically intriguing allosteric pathway distinct from benzodiazepine action; however, its location on a shared β_2_ subunit and the micromolar concentrations required for modulation represent substantial obstacles to the rational design of anxioselective agents based on this mechanism.

## 1 Introduction

Anxiety, like fear, is an adaptive defensive state that supports survival; however, when this response becomes dysregulated, it contributes to clinically significant anxiety disorders (Yamamori and Robinson, 2023). Although the neurobiology of these disorders is multifactorial and remains only partially understood, convergent evidence indicates that altered γ-aminobutyric acid (GABA)-mediated inhibition is an important component of their pathophysiology (Chhatwal and Ressler, 2007). In the adult mammalian central nervous system, GABAergic signaling is the principal mechanism of fast inhibition and is essential for maintaining the balance between excitation and inhibition required for normal network function (Kim and Hibbs, 2022). GABA acts through two main receptor classes: ionotropic GABA-A receptors, which form chloride-selective ligand-gated ion channels, and metabotropic GABA-B receptors, which signal through Gi/o proteins (Ghit et al., 2021). This review focuses on GABA-A receptors because of their central role in rapid synaptic inhibition and their long-standing relevance as pharmacological targets in anxiety treatment.

From a physiological perspective, the mechanism of GABA-A receptor activation is initiated by the binding of two GABA molecules to well-described orthosteric sites, triggering a conformational change that opens a chloride ion (Cl^−^)-selective channel (Kim and Hibbs, 2022). In mature adult neurons, the chloride equilibrium potential (ECl) is approximately −70 mV, slightly more negative than the resting membrane potential (∼ − 65 mV), because the KCC2 cotransporter extrudes Cl^−^ from the cytosol (Ben-Ari et al., 2012; Kahle and Delpire, 2016). Under these conditions, GABA-A receptor activation generally produces hyperpolarizing or shunting inhibition, thereby reducing the probability of neuronal firing. This basic mechanism underlies the action of several clinically important drug classes, including anesthetics, anticonvulsants, and anxiolytics that act through GABAergic pathways (Kim and Hibbs, 2022; Sigel et al., 2011; Rudolph and Mohler, 2004).

Against this background, this review examines whether GABA-A receptors may represent a plausible mechanistic framework for evaluating cannabinoid-related ligands with anxiolytic potential (Kahle and Delpire, 2016; Kan et al., 2024; Maillard et al., 2022). Our main point of departure is the endocannabinoid 2-arachidonoylglycerol (2-AG), for which direct modulation of GABA-A receptors has been demonstrated (Blessing et al., 2015). We also discuss selected cannabinoid-related ligands, including ∆^9^-tetrahydrocannabinol (∆^9^-THC) and cannabidiol (CBD), but only in a comparative and translational context. Importantly, the direct modulation reported for 2-AG does not justify generalization to cannabinoids as a pharmacological class (Blessing et al., 2015; Maillard et al., 2022).

A seminal study by Blessing et al. (2015) identified a functional interaction site for 2-AG on the transmembrane helix M4 of the β_2_ subunit of GABA-A receptors. Because the α_1_β_2_γ_2_ isoform is widely regarded as the predominant synaptic GABA-A receptor in the adult brain and contains the β_2_ subunit implicated in 2-AG modulation (Pertwee et al., 2010; Sigel et al., 2011), it provides a relevant molecular framework for examining this mechanism. This review integrates structural, biophysical, and pharmacological evidence related to α_1_β_2_γ_2_ receptors, while drawing on closely related human synaptic GABA-A receptor structures where appropriate (Izzo et al., 2009; Kim and Hibbs, 2022).

Specifically, this review aims to (a) examine the molecular basis of direct 2-AG interaction; (b) assess, within the benzodiazepine subtype-selectivity framework, whether modulation of α_1_-containing versus α_2_/α_3_-containing receptors could, in principle, relate to sedative versus anxiolytic outcomes (Rudolph and Mohler, 2004; McKernan et al., 2000; Low et al., 2000); and (c) identify critical gaps that limit the rational design of selective cannabinoid-inspired modulators.

## 2 GABA-A receptor: structural properties and characteristics

### 2.1 The Cys-loop superfamily and receptor architecture

GABA-A receptors belong to the superfamily of pentameric ligand-gated ion channels (pLGICs), also known as the Cys-loop receptor family (Connolly and Wafford, 2004; Olsen and Sieghart, 2008). This family also includes nicotinic acetylcholine receptors, serotonin 5-HT_3_ receptors, and strychnine-sensitive glycine receptors. Despite their functional differences, all members share the same basic quaternary organization: five subunits arranged around a central membrane-spanning pore that is selective for specific ions (Kim and Hibbs, 2022).

GABA-A receptors follow this general architecture. They form cylindrical pentamers with a central pore that crosses the membrane (Kim and Hibbs, 2022; Zhu et al., 2018). Each subunit contains a large extracellular domain (ECD) at the N-terminus, four transmembrane helices (M1–M4), and a variable intracellular domain (ICD) formed by the large cytoplasmic loop between M3 and M4 (Miller and Aricescu, 2014). The ECD contains the orthosteric GABA-binding sites, whereas the transmembrane domain (TMD) forms the ion pore and harbors binding sites for allosteric modulators, including some anesthetics and neurosteroids (Kim and Hibbs, 2022; Chen et al., 2018). By contrast, the ICD remains less well resolved structurally. In several experimental structures, parts of this region are missing or appear disordered, even though it has established roles in receptor modulation, trafficking, and interactions with synaptic anchoring proteins (Kim and Hibbs, 2022; Lorenz-Guertin and Jacob, 2017; Tretter et al., 2012). This structural flexibility continues to limit a more complete understanding of how the ICD contributes to receptor regulation. This architecture is directly relevant to the present review because the proposed 2-AG-sensitive region lies on β_2_-M4, the outermost transmembrane helix and the one most directly exposed to the surrounding lipid bilayer.

### 2.2 Subunit heterogeneity and receptor diversity

GABA-A receptors exhibit substantial molecular diversity because they are assembled from multiple subunit genes. In humans, 19 genes encode GABA-A receptor subunits, grouped into several families based on sequence homology: α_1_–α_6_, β_1_–β_3_, γ_1_–γ_3_, δ, ϵ, θ, π, and ρ_1_–ρ_3_ (Kim and Hibbs, 2022; Olsen and Sieghart, 2008). These subunits are assembled in the endoplasmic reticulum to generate a broad range of functional heteropentamers (Kim and Hibbs, 2022).

Even so, this diversity is not random. In the brain, the most common GABA-A stoichiometry is two α subunits, two β subunits, and one γ or δ subunit (Kim and Hibbs, 2022). The α_1_β_2_γ_2_ isoform is widely regarded as the predominant synaptic isoform in the central nervous system and is often estimated to account for roughly 40–50% of native GABA-A receptors (Pertwee et al., 2010; Kim and Hibbs, 2022; Belelli et al., 2025; Pirker et al., 2000).

Other receptor populations show more restricted distributions. α_2_- and α_3_-containing isoforms are enriched in specific neuronal populations and brain regions rather than being uniformly expressed throughout the CNS (Kim and Hibbs, 2022; Belelli et al., 2025; Fritschy and Mohler, 1995). Likewise, α_5_-containing receptors are particularly enriched in the hippocampus, whereas α_4_/δ-containing receptors are characteristic of the thalamus (Pertwee et al., 2010; Kim and Hibbs, 2022; Belelli et al., 2025; Fritschy and Mohler, 1995).

These differences matter because subunit composition shapes not only the receptor’s biophysical and pharmacological properties, but also its subcellular localization and physiological role. In this review, receptor diversity is therefore not introduced as background alone. It is central to the discussion because α_1_β_2_γ_2_, α_2_/α_3_-containing receptors, and extrasynaptic α_5_/δ-containing assemblies define the principal receptor populations for the cannabinoid-related mechanisms discussed later.

### 2.3 Functional segregation: phasic versus tonic inhibition

Differences in subunit composition and subcellular localization of GABA-A receptors give rise to two functionally distinct modes of inhibition: phasic and tonic (Sigel et al., 2011; Farrant and Nusser, 2005). This distinction is essential for understanding both normal circuit regulation and the effects of receptor modulation by drugs or endogenous ligands.

Phasic inhibition is the classical form of fast synaptic neurotransmission. It is mediated by GABA-A receptors concentrated at postsynaptic sites opposite GABAergic presynaptic terminals and has been extensively characterized in forebrain and cerebellar circuits, including the hippocampus, neocortex, and cerebellum (Sigel et al., 2011; Farrant and Nusser, 2005; Cherubini, 2012). These receptors typically contain a γ subunit, as in the α_1_β_2_γ_2_ isoform. They have relatively low affinity for GABA and are activated by transient high-concentration (mM) bursts of neurotransmitter released into the synaptic cleft. The result is a rapid, short-duration inhibitory postsynaptic current (IPSC) that regulates neuronal activity on millisecond timescales (Sigel et al., 2011; Farrant and Nusser, 2005).

In contrast, tonic inhibition is a more persistent form of signaling. It is mediated by receptors localized in extrasynaptic or perisynaptic regions. These receptors often contain a δ subunit (e.g., α_4_βδ or α_6_βδ) or, in some regions such as the hippocampus, α_5_-containing receptors. Because they display high affinity for GABA, they can be activated by low ambient concentrations of transmitter (μM or lower) that diffuse beyond the synaptic cleft. Their activation generates a sustained Cl^−^ conductance, or inhibitory tone, that helps regulate overall neuronal excitability and firing threshold (Pertwee et al., 2010; Farrant and Nusser, 2005; Brickley and Mody, 2013).

This functional distinction is especially relevant for the present review because any direct modulation of GABA-A receptors by cannabinoid-related ligands must ultimately be interpreted in relation to synaptic versus extrasynaptic receptor populations.

### 2.4 Pathophysiological relevance for anxiety and neurological disorders

Due to their central role in controlling neuronal excitability, dysfunction of the GABA-A system has been implicated in a wide range of neurological and psychiatric disorders. Altered excitation-inhibition balance is a recurring feature of conditions such as epilepsy, anxiety disorders, insomnia, schizophrenia, and depression. For this reason, GABA-A receptors remain major pharmacological targets in the CNS and continue to motivate the development of anxiolytic, sedative, hypnotic, anticonvulsant, and general anesthetic drugs (Kim and Hibbs, 2022; Laverty et al., 2019; Rudolph and Mohler, 2004; Korpi and Sinkkonen, 2006).

Their relevance to anxiety is particularly well established. Benzodiazepines, the best-known anxiolytic drugs, act primarily by potentiating GABAergic inhibition (Rudolph and Mohler, 2004). Importantly, anxiolytic effects are mediated mainly by α_2_-containing receptors, with contributions from α_3_-containing receptors, whereas sedative effects are associated predominantly with α_1_-containing receptors (McKernan et al., 2000; Low et al., 2000). This subtype-dependent dissociation is one reason why GABA-A receptors remain central to efforts aimed at developing safer anxiolytic drugs.

Genetic evidence further supports the clinical importance of this receptor system. Variants in genes encoding GABA-A receptor subunits, including *GABRA1*, *GABRB2*, and *GABRG2*, are associated with neurological disorders, particularly epileptic and developmental epileptic encephalopathies (Ghit et al., 2021; Masiulis et al., 2019; Hernandez et al., 2021; Maillard et al., 2022; Kang and Macdonald, 2017). Although the direct relationship between these mutations and anxiety is less clearly established, association studies have linked *GABRA2* variants to anxiety-related phenotypes and alcohol- related behaviors (Low et al., 2000; Connolly and Wafford, 2004; Enoch et al., 2010; Duka et al., 2015). Functionally, these variants may alter channel gating and/or disrupt receptor assembly, maturation, or trafficking to the neuronal membrane.

This genotype–phenotype relationship underscores the importance of maintaining a functional population of GABA-A receptors at the plasma membrane for normal brain function. It also provides a rationale for investigating how exogenous ligands may modulate specific receptor subtypes to restore neuronal homeostasis.

Taken together, these structural, subunit-specific, and functional features make the α_1_β_2_γ_2_ receptor the most useful framework for evaluating direct 2-AG-sensitive modulation, while also highlighting the receptor populations that define the physiological and translational limits of this mechanism.

## 3 High-resolution structural architecture of the α_1_β_2_γ_2_ isoform

### 3.1 Advances in Cryo-EM and protein structure determination

Advances in single-particle cryo-electron microscopy (cryo-EM) have transformed structural analysis of GABA-A receptors over the past decade (Kim and Hibbs, 2022). Before these developments, structural interpretation depended largely on homology-based models derived from related Cys-loop receptors. Those models were useful for defining overall architecture, but they lacked the resolution needed to localize pharmacologically relevant cavities or to examine subunit-specific determinants of allosteric modulation in detail.

Cryo-EM has removed many of the constraints that previously limited structural work on membrane proteins, making it possible to solve intact GABA-A receptors in defined conformational states and, in several cases, in membrane-mimetic environments (Kim and Hibbs, 2022). This is especially relevant here because the proposed cannabinoid-sensitive region lies on β_2_-M4, a transmembrane helix exposed to the surrounding lipid bilayer. Structures obtained under native-like conditions are often more informative than detergent- only preparations when discussing lipophilic ligands and membrane-dependent mechanisms (Kim and Hibbs, 2022; Notti and Walz, 2023). They also provide a strong starting point for molecular-dynamics studies of receptor motions and ligand–receptor interactions at atomic resolution.

### 3.2 Detailed analysis of PDB structures for the human α_1_β_2_γ_2_ isoform

The availability of multiple structures in the Protein Data Bank (PDB) provides a practical framework for analyzing the human α_1_β_2_γ_2_ receptor background. These structures are not interchangeable: they differ in subunit composition, conformational state, ligand occupancy, and overall proximity to native receptor assemblies. Those differences matter here because interpretation of cannabinoid-related modulation depends on how closely each template reflects β_2_-containing receptors.

Among currently available datasets, recently resolved native human-brain assemblies provide especially useful context for physiological organization and membrane-proximal interactions (Zhou et al., 2025). Recombinant human α_1_β_2_γ_2_ structures remain highly valuable for defining canonical pharmacological interfaces and gating-related conformations (Zhu et al., 2018; Miller and Aricescu, 2014). Other influential structures, including those that contain β_3_ instead of β_2_, are also informative, but they should be interpreted cautiously for cannabinoid-focused questions because the site proposed by Sigel et al. was mapped specifically to β_2_ residues in M4 (Blessing et al., 2015; Laverty et al., 2019; Masiulis et al., 2019). In that sense, β_3_-containing templates are better viewed as complementary frameworks than as direct mechanistic equivalents for β_2_-M4-sensitive modulation.

### 3.3 Key structural domains

The architecture of the α_1_β_2_γ_2_ receptor can be summarized in three major domains:

(1) *Extracellular domain (ECD)*: This region contains the orthosteric GABA-binding sites and also contributes to recognition of modulators such as benzodiazepines. These pockets are formed at subunit interfaces between principal (+) and complementary (−) faces (Kim and Hibbs, 2022).
(2) *Transmembrane domain (TMD)*: Each subunit contributes four helices (M1–M4). The five M2 helices line the pore and contain key elements for gating and ion selectivity. At the same time, intersubunit transmembrane interfaces generate cavities that host several classes of allosteric modulators (Sigel et al., 2011; Masiulis et al., 2019; Puthenkalam et al., 2016). In the present review, the TMD is especially important because the proposed 2-AG-sensitive region lies on β_2_-M4.
(3) *Intracellular domain (ICD)*: This region is defined largely by the M3–M4 cytoplasmic loop and remains the least well resolved part of the receptor in many high-resolution models (Kim and Hibbs, 2022; Laverty et al., 2019; Zhu et al., 2018). Even so, it plays established roles in trafficking, synaptic targeting, and regulatory protein interactions (Lorenz-Guertin and Jacob, 2017; Tretter et al., 2012).
Together, these domains provide the structural basis for interpreting both canonical pharmacological sites and candidate non-canonical lipid-proximal mechanisms.

### 3.4 Post-translational modifications and auxiliary subunits

The native complexity of the receptor extends beyond its pentameric core. Post-translational modifications and association with auxiliary proteins are important for biogenesis, membrane delivery, and synaptic organization; for that reason, they also shape how the proposed cannabinoid-sensitive region should be interpreted in native receptor assemblies.

One important example is glycosylation. GABA-A receptor subunits are glycosylated at conserved sites within the ECD. Cryo-EM structures such as 9CRS resolve glycan densities at specific residues of the α_1_β_2_γ_2_ isoform, including N111 in α_1_, N77 and N141 in β_2_, and N208 in γ_2_ (Chen et al., 2018). These modifications contribute to proper subunit folding, pentamer assembly in the endoplasmic reticulum, and subsequent trafficking to the cell surface (Lo et al., 2010). In that sense, the structural state captured in high-resolution models reflects not only subunit arrangement, but also post-translational maturation.

A second layer of complexity comes from auxiliary and associated proteins. GABA-A receptors are increasingly understood not as isolated pentamers, but as parts of larger macromolecular assemblies. Proteomic analyses of receptors purified from human brain have identified several GABA-A-associated proteins, including the synaptic adhesion molecule neuroligin-2 and GARLH4 (also known as LHFPL4) (Chen et al., 2018; Cherubini, 2012). Subsequent studies have shown that LHFPL4 is enriched at inhibitory synapses, interacts closely with receptor subunits, and contributes to clustering and function of GABA-A receptors in hippocampal pyramidal neurons (Cherubini, 2012).

This point is directly relevant to the present review because the proposed cannabinoid-sensitive site lies in the transmembrane domain (Blessing et al., 2015), and LHFPL4 is a tetraspanin that associates closely with the receptor in this same region. Its presence could therefore influence the conformation, accessibility, or dynamics of the proposed site in native receptor complexes. For that reason, structural or computational analyses based on the pentameric core remain useful as a starting point, but they are unlikely to capture the full complexity of the native receptor environment.

These observations reinforce the idea that the human α_1_β_2_γ_2_ receptor provides a useful structural framework for studying canonical pharmacological cavities and for evaluating the proposed non-canonical β_2_-M4-sensitive mechanism of 2-AG. At the same time, this framework must be interpreted within the broader context of native receptor organization, a point that becomes especially relevant in anxiety research, where therapeutic and adverse effects of GABA-A receptor modulation are strongly shaped by the receptor populations that are engaged.

## 4 The pharmacological landscape: binding sites and modulators

**Figure 1:**
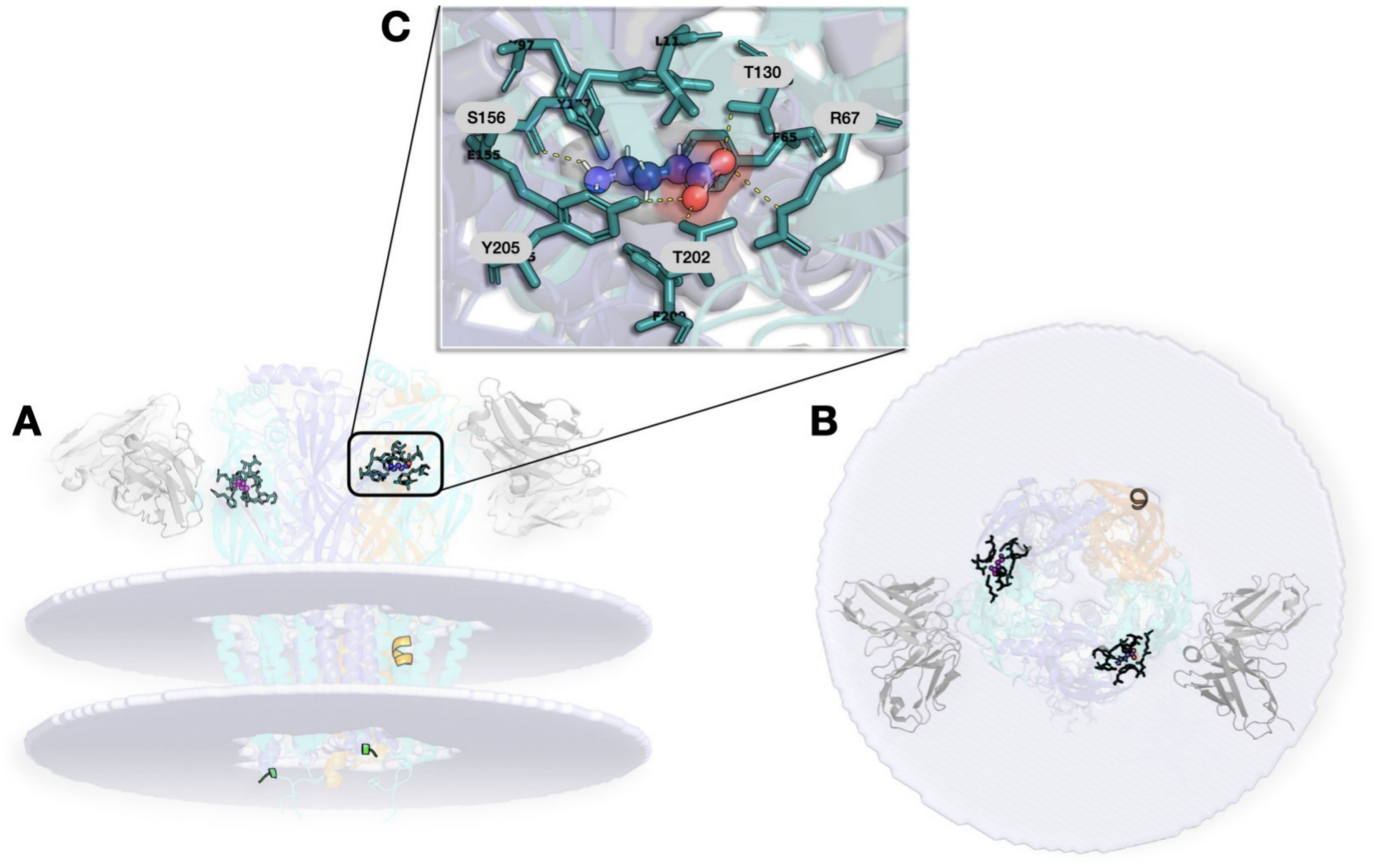
Structural architecture of the native human brain GABA-A receptor (PDB ID: 9CRS). **(A)** Lateral view of the receptor complex rendered in a membrane model, with the extracellular domain oriented upwards and the cytoplasmic side downwards. Fiducial Fab 1F4 fragments are bound to the extracellular domain. Conserved N-linked glycosylation sites resolved in the structure include N111 in α_1_, N77 and N141 in β_2_, and N208 in γ_2_. **(B)** Extracellular top view showing the pseudo-symmetrical β_2_-α_1_-β_2_-α_1_-γ_2_ pentameric arrangement around the central chloride ion pore. The subunits correspond to chains A and C (β_2_), B and D (α_1_), and E (γ_2_). **(C)** Close-up of the orthosteric GABA-binding site located at the β(+)/α(−) interface, with γ-aminobutyric acid (GABA) shown within the recognition pocket. This structure corresponds to a native human α_1_β_2_γ_2_ receptor assembly resolved from human brain tissue and provides the most directly relevant structural framework for evaluating the proposed β_2_-M4 cannabinoid-sensitive mechanism.

The GABA-A receptor is one of the most pharmacologically complex targets in the CNS, with multiple binding sites that enable diverse and fine-tuned modulation of receptor activity (Kim and Hibbs, 2022; Goldschen-Ohm, 2022; Hibbs and Gouaux, 2011; Masiulis et al., 2019). Among its many assemblies, the α_1_β_2_γ_2_ isoform is especially relevant because it is both highly abundant in the brain and one of the best-characterized pharmacological backgrounds (Pertwee et al., 2010; Kim and Hibbs, 2022). Figure 1 illustrates the structural architecture of the native human-brain α_1_β_2_γ_2_ GABA-A receptor (PDB ID: 9CRS) (Chen et al., 2018).

### 4.1 Mapping binding cavities in the receptor

The pentameric architecture of the receptor generates several ligand-binding cavities, each associated with distinct classes of modulators and functional consequences (Table 1).

**Table 1:** Resolved PDB structures relevant to structural analysis of human synaptic GABA-A receptors.

PDB	Assembly	Structural state	Resolution	Bound ligands/preparation context	References
9CRS	Native human β_2_-α_1_-β_2_-α_1_-γ_2_	GABA-bound, active-like	2.90 A°	Native human brain receptor; Fab 1F4	Zhou et al. (2025)
9DRX	Native human β_2_-α_1_-β_2_-α_1_-γ_2_	GABA/lamotrigin2e. bound	2.95 A°	Native human brain receptor; lamotrigine present	Zhou et al. (2025)
9CXC	Native human β_3_-α_1_-γ_2_-β_2_-α_2_	Basal/ligand-free mixed assembly	3.30 A°	Native human brain receptor; mixed α/β composition	Zhou et al. (2025)
6X3X	Recombinant α_1_β_2_γ_2_	GABA-bound, active-like	2.92 A°	GABA + diazepam	Kim et al. (2020)
6HUP	Recombinant α_1_β_3_γ_2_L	Desensitized	3.58 A°	GABA + diazepam + megabody Mb38	Laverty et al. (2019)
6I53	Recombinant α_1_β_3_γ_2_L	Desensitized	3.20 A°	Apo + megabody Mb38; lipid nanodisc preparation	Laverty et al. (2019)

Structures containing β_3_ rather than β_2_ are included for structural and historical context, but their direct applicability to the proposed 2-AG-sensitive β_2_-M4 mechanism is limited unless the β_3_-to-β_2_ difference is explicitly addressed computationally.

The orthosteric GABA sites are located in the extracellular domain (ECD) at interfaces between the principal face (+) of a β subunit and the complementary face (−) of an α subunit. Binding is mediated by an aromatic cluster formed by tyrosine and phenylalanine residues, together with key electrostatic interactions and hydrogen bonds (Kim and Hibbs, 2022). Competitive antagonists such as bicuculline and gabazine compete with GABA at this same site (Pertwee et al., 2010).

The benzodiazepine (BZD) site lies at a homologous extracellular interface, but in this case between the principal face (+) of an α subunit and the complementary face (−) of a γ subunit (Laverty et al., 2019). Sensitivity to classical BZDs such as diazepam requires receptors containing α_1_, α_2_, α_3_, or α_5_ together with a γ subunit, and a conserved histidine (H102) in α_1_ is a key determinant of high-affinity binding (Kim and Hibbs, 2022; Laverty et al., 2019). By contrast, receptors containing α_4_ or α_6_ subunits are largely insensitive to classical BZDs (Laverty et al., 2019).

Within the transmembrane domain (TMD), intersubunit cavities serve as binding sites for intravenous anesthetics. Etomidate shows preference for interfaces containing β_2_ or β_3_ subunits and binds within a cavity formed by β-M3 and α-M1 helices, whereas propofol appears less selective and can interact with multiple β/α interfaces in the TMD (Kim and Hibbs, 2022). Phenobarbital does not appear to depend on a single high-affinity pocket, but rather on more distributed effects on receptor conformational dynamics (Ghit et al., 2021; Kim and Hibbs, 2022).

The channel pore, lined by M2 helices, provides another pharmacologically relevant site. Non-competitive blockers such as picrotoxin bind within the pore, physically occluding chloride flux and preventing normal channel conduction (Ghit et al., 2021).

**Figure 2:**
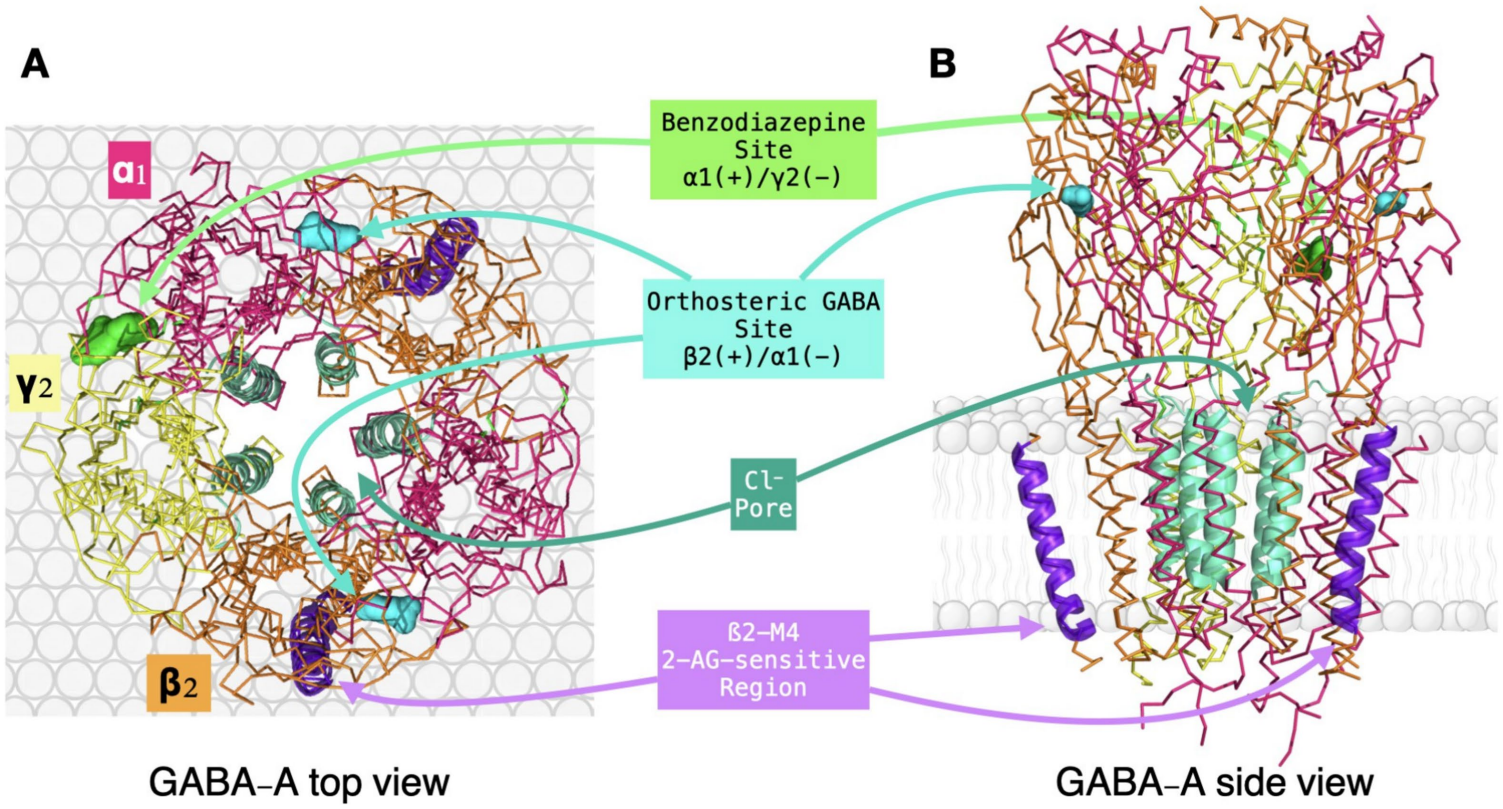
Structural summary of canonical and proposed non-canonical interaction regions in the native human α_1_β_2_γ_2_ GABA-A receptor based on structure 9CRS. **(A)** Top view of the pentameric receptor showing the arrangement of subunits and the main extracellular pharmacological interfaces, including the two orthosteric GABA-binding sites at β_2_(+)/α_1_(−) interfaces and the canonical benzodiazepine-binding site at the α_1_(+)/γ_2_(−) interface. **(B)** Side view of the receptor embedded in the lipid bilayer, illustrating the chloride-conducting pore and the β_2_-M4 region proposed to participate in 2-AG-sensitive modulation. This figure summarizes the structural features discussed in Sections 2.2–2.4 and distinguishes canonical pharmacological sites from the proposed non-canonical β_2_-M4-sensitive mechanism. Color code: α_1_ subunits, magenta; β_2_ subunits, orange; γ_2_ subunit, yellow; β_2_-M4 region, purple; Cl^−^ pore, dark green; GABA sites, cyan; benzodiazepine site, lime green.

Together, these canonical sites illustrate the pharmacological versatility of the α_1_β_2_γ_2_ receptor and the existence of multiple allosteric pathways that modulate receptor function. Ligands acting in the ECD, such as GABA and benzodiazepines, initiate conformational changes that propagate toward the gate, whereas ligands acting in the TMD can influence channel behavior more directly through local rearrangements of pore-associated helices. Table 2 summarizes representative pharmacological tools acting on α_1_-containing GABA-A receptors and provides a benchmark against which the proposed non-canonical β_2_-M4-sensitive mechanism can be conceptually compared. Against this established framework, the next question is whether the endocannabinoid 2-arachidonoylglycerol (2-AG) engages a distinct non-canonical modulatory site with plausible relevance for anxiolytic drug development (Figure 2).

**Table 2:** Representative benzodiazepine-site pharmacological tools used to benchmark α_1_- versus α_2_/α_3_-containing GABA-A receptor pharmacology.

Compound	Type	Relative subtype profile	Main relevance in this review	References
Diazepam	PAM	Non-selective across benzodiazepine-sensitive α_1_/α_2_/α_3_/α_5_ receptors	Classical benchmark for broad benzodiazepine pharmacology	Rudolph and Mohler (2004) and Atack (2010)
Zolpidem	PAM	α1-preferring	Sedative/hypnotic reference compound	Sanna et al. (2009)
βCCt	Antagonist/NAM-like tool at BZD site	α_1_-preferring	Tool compound to probe α1-linked effects	Rowlett et al. (2005)
SL651498	PAM	α_2_/α_3_-preferring over α_1_	Prototype anxioselective-like ligand	Atack (2010) and Griebel et al. (2001)
L-838,417	PAM	α_2_/α_3_/α_5_-active, α_1_-sparing	Classic tool compound for the anxioselectivity hypothesis	McKernan et al. (2000) and Morris et al. (2009)

This table summarizes the classical subtype-selectivity framework that underlies the anxioselectivity hypothesis, against which the translational potential of the proposed β_2_-M4 cannabinoid-sensitive mechanism is evaluated.

## 5 Cannabinoid interactions with GABA-A receptors: a novel modulatory axis

### 5.1 Evidence for direct, non-canonical interaction

Electrophysiological studies indicate that some cannabinoid-related ligands can directly modulate GABA- A receptor-mediated currents, but this effect is not uniform across compounds or experimental systems (Sigel et al., 2011; Bakas et al., 2017). The clearest example remains 2-arachidonoylglycerol (2-AG). Sigel and colleagues showed potentiation of recombinant α_1_β_2_γ_2_ currents and linked this effect to residues in the M4 helix of the β_2_ subunit (Sigel et al., 2011). However, potentiation was observed at relatively high concentrations (approximately 10–30 μM), above many reported endogenous ranges in tissue and biofluids (Kucera et al., 2010; Kratz et al., 2021).

The situation is less straightforward for other cannabinoid-related ligands. ∆^9^-THC shows dose- and context-dependent effects on anxiety, with low-dose anxiolytic-like and high-dose anxiogenic or sedative profiles mainly interpreted in CB1-centered paradigms (Sharpe et al., 2020; Rey et al., 2012; Childs et al., 2019). Cannabidiol (CBD), in contrast, has a broader anxiolytic pharmacology and has also been reported to modulate selected GABA-A receptor subtypes in electrophysiological assays (Blessing et al., 2015; Bakas et al., 2017). Even so, current direct evidence remains strongest for 2-AG and does not justify extending a single mechanism to cannabinoid-related ligands as a whole.

The functional dichotomy reported in some *in vitro* paradigms—suppression of phasic inhibition together with potentiation of tonic inhibition—requires careful interpretation (Golovko et al., 2014). The pattern is interesting, but its *in vivo* relevance remains uncertain. It is also not established that this dual effect arises uniformly from direct receptor-level interaction. At least part of the effect may reflect indirect presynaptic CB1 mechanisms that alter GABA release (Katona et al., 1999; Hoffman and Lupica, 2000).

### 5.2 A putative binding site in the transmembrane domain

The proposed direct-interaction site is a central mechanistic issue in this field. Electrophysiology and mutagenesis indicate that 2-AG potentiates recombinant GABA currents and that this effect depends on residues within the M4 segment of the β_2_ subunit (Sigel et al., 2011; Baur et al., 2013). This observation places the putative cannabinoid- sensitive region in an unusual position: not at canonical extracellular or pore-associated sites, but on M4, the outermost transmembrane helix and the one most directly exposed to the lipid bilayer.

That location raises the possibility that site access occurs, at least in part, through the membrane itself. Given the lipophilic character of 2-AG and related ligands, a lateral route through the bilayer is plausible. This “through-the-lipids” hypothesis is chemically intuitive, but it remains inferential and still lacks direct structural confirmation (Rimmerman et al., 2007; Maccarrone, 2007).

This point also matters for translation. Because the proposed site is located on the β_2_ subunit rather than on α subunits, it does not map directly onto subtype-selectivity logic that has traditionally guided anxiolytic GABA-A drug development. Any therapeutic implication therefore has to be judged against that established pharmacological framework rather than assumed from it.

### 5.3 Functional dichotomy: synaptic versus extrasynaptic modulation

Cannabinoid-related modulation of GABA-A receptors does not appear to produce a single uniform functional outcome. Depending on context, these ligands may affect synaptic and extrasynaptic receptor populations differently. In some preparations, cannabinoid-related compounds reduce phasic inhibition while enhancing tonic inhibition, effectively shifting both modes in opposite directions (Golovko et al., 2014; Golovko et al., 2014).

This dual behavior complicates network-level interpretation. Reduced phasic inhibition may increase excitability in some settings, whereas enhanced tonic inhibition may have the opposite effect. In anxiety- relevant circuits, net outcome likely depends on cell type, microcircuit position, and broader network state.

The lipid-access hypothesis provides one way to frame this complexity. Lateral diffusion through the bilayer is compatible with structural evidence that transmembrane cavities can host lipophilic modulators (Kim and Hibbs, 2022; Puthenkalam et al., 2016). Still, extending this idea to explain synaptic-versus-extrasynaptic divergence goes beyond current direct evidence. Data linking inhibitory synapses to lipid microdomains that materially shift GABA-A pharmacology remain limited, although raft-like localization has been reported in cerebellar granule cells and broader reviews emphasize persistent uncertainty about lipid contributions to postsynaptic organization (Dalskov et al., 2005; Westra et al., 2021).

There is also no direct evidence that local lipid composition can shift M4 conformation enough to reverse the direction of allosteric modulation, changing a potentiating effect into an inhibitory one. For now, that remains a plausible interpretation rather than an established mechanism. Testing it will require direct biophysical studies and computational analyses in membrane systems with controlled lipid composition.

### 5.4 Neuroplasticity and long-term effects

Longer-term cannabinoid exposure raises a related but distinct issue. Deshpande and colleagues reported reduced mIPSC amplitude and decreased surface density of GABA-A receptor β_2_/β_3_ subunits after 24 h of WIN 55,212–2 treatment in hippocampal cultures (Deshpande et al., 2012). These findings are important but do not directly demonstrate interaction with the proposed β_2_-M4 site. Because WIN 55,212–2 is a potent CB1 agonist, a parsimonious interpretation is that at least part of this effect reflects neuroadaptation secondary to sustained CB1 signaling and altered presynaptic GABA release (Katona et al., 1999; Hoffman and Lupica, 2000).

This distinction separates acute receptor-level modulation from longer-term circuit and trafficking adaptations. Future work should explicitly distinguish direct effects at the proposed β_2_-M4 site from indirect CB1-driven mechanisms, ideally using selective ligands, subtype-focused pharmacology, and receptor-specific genetic perturbation strategies.

Overall, current evidence suggests that cannabinoid-sensitive modulation of GABA-A receptors cannot be interpreted independently of receptor assembly, membrane context, and synaptic organization.

## 6 The cellular environment: biogenesis, trafficking, and the neuronal membrane

Any attempt to interpret interactions between GABA-A receptors and cannabinoid-related ligands must account for cellular context. The receptor is not simply a pentamer embedded in a passive bilayer. Its behavior depends on assembly, trafficking to the membrane, synaptic stabilization, and local lipid composition. These factors can influence ligand access, receptor conformation, and the eventual functional outcome of modulation.

Three aspects are especially relevant here. First, membrane lipids such as cholesterol and PIP2 can influence both ligand partitioning and the conformation of the M4 region where the proposed 2-AG- sensitive site is located (Blessing et al., 2015; Kim and Hibbs, 2022). Second, prolonged cannabinoid exposure can alter surface expression and inhibitory signaling in receptors containing β_2_/β_3_ subunits (Deshpande et al., 2012). Third, auxiliary proteins such as LHFPL4, which participate in synaptic clustering, associate near transmembrane regions and may influence accessibility of the β_2_-M4 zone (Davenport et al., 2017). This section integrates these elements to place the proposed cannabinoid-receptor interaction in a more physiological framework.

### 6.1 Receptor biogenesis and endoplasmic reticulum quality control

The life cycle of a functional GABA-A receptor begins in the endoplasmic reticulum (ER), where subunits are synthesized, folded, post-translationally modified, and assembled with the appropriate stoichiometry (Sarto-Jackson and Sieghart, 2008). The ER membrane complex also contributes to membrane insertion and early receptor biogenesis (Whittsette et al., 2025). ER chaperones then support productive folding and assembly.

Subunits that fail to fold or assemble correctly are retained and routed to ER-associated degradation (ERAD), returning them to the cytosol for proteasomal disposal (Macdonald and Kang, 2013; Sarto-Jackson and Sieghart, 2008). Many epilepsy-associated variants disrupt these steps, causing ER retention and reduced receptor surface expression (Macdonald and Kang, 2013). Thus, receptor availability at the membrane is constrained well before any extracellular ligand binds.

### 6.2 Trafficking and synaptic clustering

Only correctly assembled pentamers can exit the ER, transit through the Golgi, and reach the plasma membrane (Sarto-Jackson and Sieghart, 2008; Bogdanov et al., 2006). Newly inserted receptors may first appear extrasynaptically before redistributing laterally within the membrane. For phasic signaling populations, this is followed by recruitment and stabilization at inhibitory synapses through scaffolding complexes centered on gephyrin (Tretter et al., 2012; Essrich et al., 1998). By forming a submembranous lattice, gephyrin limits lateral diffusion and supports receptor accumulation opposite presynaptic GABAergic terminals.

This process is not governed by gephyrin alone. Native receptor assemblies also include associated proteins such as GARLH4/LHFPL4, which are relevant for clustering of α_1_-containing receptors (Zhou et al., 2025; Davenport et al., 2017). Because LHFPL4 associates near transmembrane segments, it may influence accessibility or local conformation of the proposed cannabinoid-sensitive region on β_2_-M4.

Final anchoring also depends strongly on the γ_2_ subunit. Loss of γ_2_ disrupts synaptic clustering of major GABA-A receptor populations and gephyrin organization (Essrich et al., 1998). Although the α_2_ loop contains a direct gephyrin-interaction motif, α_1_-containing receptors appear to rely more heavily on γ_2_-dependent mechanisms for stable synaptic localization (Puthenkalam et al., 2016; Essrich et al., 1998). This distinction matters because the receptor population central to this review is defined not only by subunit composition, but also by its spatial positioning within the synaptic membrane.

### 6.3 Neuronal membrane lipid composition and relevance for cannabinoid modulation

The neuronal membrane is neither uniform nor symmetric. It contains microdomains with distinct lipid composition, and this heterogeneity can influence receptor localization, dynamics, and pharmacology (Borroni et al., 2016). GABA-A receptors have been observed in raft-like microdomains in cerebellar granule cells (Dalskov et al., 2005), and recent work suggests that astrocytic cholesterol can shift receptor lipid-domain association and functional behavior (Yuan et al., 2025). Cholesterol therefore appears to shape the membrane context of GABA-A signaling, although its role in stabilizing specific α_1_β_2_γ_2_ conformations remains unresolved.

PIP2 adds a further layer of complexity. In nanodisc-reconstituted α_1_β_3_γ_2_ structures, PIP2 interacts with positively charged juxtamembrane cavities in α_1_ subunits (Kim and Hibbs, 2022; Laverty et al., 2019). This interaction has not yet been directly resolved in α_1_β_2_γ_2_ receptors, but conservation of key α_1_ residues supports the possibility of related lipid coupling in that isoform.

For cannabinoid-related ligands, this matters because their marked lipophilicity makes membrane partitioning part of the mechanism rather than simple background. Differences in local membrane composition may affect lateral diffusion in the bilayer and access to the proposed β_2_-M4 region. It is therefore plausible that synaptic and extrasynaptic membranes do not provide equivalent environments for cannabinoid-sensitive modulation. Even so, the idea that lipid context could reverse the direction of modulation, from potentiating to inhibitory, remains hypothetical. This possibility still requires direct testing through biophysical approaches and molecular-dynamics studies in membrane models that better approximate neuronal lipid composition (Ingólfsson et al., 2017).

## 7 Discussion: clinical context and therapeutic implications

Research on GABA-A receptors has long been linked to the treatment of neurological and psychiatric disorders. In the present context, the translational question is not only whether cannabinoid-related ligands can modulate GABA-A receptors, but whether the proposed β_2_-M4-sensitive mechanism could plausibly support clinically useful anxiolysis without reproducing the known liabilities of classical GABAergic drugs.

### 7.1 GABA-A modulators in anxiety treatment

Positive allosteric modulators (PAMs), especially benzodiazepines, have been central to anxiety treatment for decades because they potentiate GABAergic inhibition and usually produce rapid symptom relief (Rudolph and Mohler, 2004; Rudolph and Knoflach, 2011). Their clinical utility, however, is limited by sedation, cognitive impairment, motor incoordination, tolerance, dependence, and withdrawal with prolonged use (Rudolph and Mohler, 2004; Engin et al., 2023). These limitations are the main reason why the field moved from broad potentiation toward attempts at subtype-informed modulation.

### 7.2 The subtype selectivity hypothesis for anxiolysis

The subtype-selectivity hypothesis proposes that benzodiazepine effects map to receptor populations defined by α-subunit identity. In this framework, sedative and amnestic effects are linked mainly to α_1_-containing receptors, whereas anxiolytic effects are associated primarily with α_2_- and α_3_-containing receptors (McKernan et al., 2000; Low et al., 2000; Rudolph and Knoflach, 2011). Receptors containing α_5_ are more strongly associated with cognitive processes (Rudolph and Knoflach, 2011). This framework has clear implications for drug design: in principle, compounds favoring α_2_/α_3_ over α_1_ could preserve anxiolysis while reducing sedation. It is also the main benchmark against which cannabinoid-related mechanisms have to be judged. Here a major limitation becomes apparent. Benzodiazepines achieve selectivity at the canonical α/γ extracellular interface (Sigel and Ernst, 2018), whereas the putative 2-AG-sensitive site is located on the M4 helix of the β_2_ subunit (Sigel et al., 2011; Baur et al., 2013). Because β_2_ is shared across receptor populations associated with both sedation and anxiolysis, a ligand acting at this site would likely modulate multiple receptor classes simultaneously. This does not rule out therapeutic relevance, but it makes straightforward anxioselective translation much less likely.

### 7.3 Review of key clinical trials and novel drugs

Recent clinical programs illustrate both the appeal and the limitations of subtype-informed GABA-A pharmacology. Darigabat (PF-06372865), an α_2_/α_3_/α_5_-preferring PAM, showed encouraging preclinical and early translational signals, including effects in challenge paradigms, but did not separate from placebo on the primary endpoint in a published phase 2 generalized anxiety disorder trial (Nickolls et al., 2018; Simen et al., 2019; Perucca et al., 2023).

Basmisanil, a selective negative allosteric modulator at α_5_-containing receptors, demonstrated target engagement but failed to deliver clear efficacy in phase 2 testing for cognitive outcomes in Down syndrome (Goeldner et al., 2022; Hipp et al., 2021). These results reinforce a broader pattern: plausible receptor-level mechanisms do not automatically translate into clinical efficacy.

Neurosteroids offer a contrasting example. Brexanolone and, later, zuranolone showed that strong GABAergic modulation can yield clinically meaningful outcomes in postpartum depression, including relatively rapid effects compared with conventional antidepressant timelines (Meltzer-Brody et al., 2018; U.S. Food and Drug Administration (FDA), 2023; Deligiannidis and Clayton, 2023). Although these agents are not subtype-selective in the benzodiazepine sense, they confirm that GABAergic pharmacology remains therapeutically relevant. Against this background, cannabinoid-related mechanisms must be evaluated by translational plausibility rather than mechanistic novelty alone.

### 7.4 Limited clinical evidence and risks of cannabinoids for anxiety disorders

Current human evidence does not yet support phytocannabinoids as validated anxiolytic treatments in routine practice. CBD has shown anxiolytic effects in acute paradigms such as simulated public speaking, but evidence in diagnosed anxiety disorders remains limited and methodologically heterogeneous (Bergamaschi et al., 2011; Blessing et al., 2015). For ∆^9^-THC, the profile is less favorable: low doses may reduce subjective stress in some settings, whereas higher doses can increase anxiety and distress (Childs et al., 2019; Sharpe et al., 2020).

Population-level evidence also warrants caution. Meta-analytic and longitudinal studies suggest a modest but significant association between cannabis exposure and adverse anxiety outcomes, with effect size and direction varying by age, exposure pattern, and vulnerability factors (Kedzior and Laeber, 2014; Xue et al., 2020; Otten et al., 2016). In parallel, stress– endocannabinoid interactions indicate broader circuit-level complexity that cannot be reduced to a single receptor mechanism (Morena et al., 2016).

Taken together, available data do not support a strong clinical case for phytocannabinoids as anxiolytics. Interest in cannabinoid-related modulation of GABA-A receptors remains justified at the mechanistic level, but therapeutic claims should remain clinically conservative.

## 8 Critical knowledge gaps and future directions

Despite sustained mechanistic interest, several central questions remain unresolved. First, physiological relevance is still uncertain. Direct potentiation by 2-AG is typically reported at concentrations around 10–30 μM, which are often above many endogenous measurements in tissue and biofluids (Sigel et al., 2011; Kucera et al., 2010). It therefore remains unclear whether this mechanism is broadly active *in vivo* or instead becomes relevant only under specific pathological or stress-related conditions (Morena et al., 2016).

Second, the molecular basis of the reported phasic-versus-tonic dichotomy remains incompletely defined. Although suppression of phasic inhibition together with enhancement of tonic inhibition has been described in some preparations, structural validation is lacking, and indirect circuit mechanisms may contribute substantially (Golovko et al., 2014; Golovko et al., 2014).

Third, subtype selectivity remains a major obstacle. Because the proposed site lies on β_2_, ligand action at this region may simultaneously affect receptor populations linked to distinct behavioral outcomes. Comparative structural and functional studies across α_1_β_2_γ_2_, α_2_β_2_γ_2_, and α_3_β_2_γ_2_ backgrounds are needed to test whether subtle local differences can produce meaningful functional bias.

Fourth, the role of membrane context is unresolved. The lipid-access hypothesis is plausible but still lacks direct experimental confirmation. It remains unknown whether realistic differences in membrane composition are sufficient to alter ligand orientation, access, or efficacy at the M4 region (Dalskov et al., 2005; Ingólfsson et al., 2017).

Finally, the largest gap is translational. There is currently no robust clinical evidence that direct GABA-A modulation by cannabinoid-related ligands contributes materially to anxiolytic efficacy in humans. Future translational work will require clear pharmacological dissection of CB1-mediated versus direct GABA-A effects, supported by target-engagement biomarkers and mechanism-focused study designs. Taken together, these gaps highlight both the promise and the limitations of the proposed β_2_-M4-sensitive mechanism. It may represent a non-canonical allosteric pathway, but functional selectivity and clinical relevance remain unproven.

## 9 Conclusion

Direct modulation of GABA-A receptors by cannabinoid-related ligands points to a non-canonical regulatory pathway that is mechanistically distinct from classical orthosteric, benzodiazepine, and anesthetic mechanisms. Across the compounds reviewed here, the strongest direct evidence remains for 2-arachidonoylglycerol (2-AG), with functional and mutational data linking modulation to residues in the M4 segment of the β_2_ subunit (Sigel et al., 2011; Baur et al., 2013). In this context, the human α_1_β_2_γ_2_ receptor remains a useful structural and pharmacological framework.

Its translational significance, however, is still uncertain. The concentrations of 2-AG required to observe direct potentiation are often higher than many reported physiological ranges, and its *in vivo* relevance remains unresolved (Sigel et al., 2011; Kucera et al., 2010). A second conceptual limitation is that the proposed site is located on β_2_, a subunit shared by receptor populations associated with both sedative and anxiolytic phenotypes in the classical framework.

Current evidence also indicates strong context dependence: cannabinoid effects vary with ligand identity, concentration, receptor subtype, and cellular environment. For that reason, the direct modulation described for 2-AG cannot be generalized to cannabinoids as a pharmacological class. Compounds such as ∆^9^-THC and CBD remain important in comparative and translational discussion, but mechanistic equivalence with 2-AG at the proposed β_2_-M4 region has not been established. Overall, the proposed β_2_-M4-sensitive mechanism is best viewed as a promising mechanistic hypothesis rather than as a validated route to anxioselective therapy. Its main value at present is conceptual: it broadens the framework for GABA-A modulation and defines concrete structural, physiological, and translational questions for future studies.
